# Application of Light Metal Alloy EN AW 6063 to Vehicle Frame Construction with an Innovated Steering Mechanism

**DOI:** 10.3390/ma13040817

**Published:** 2020-02-11

**Authors:** Miroslav Blatnický, Milan Sága, Ján Dižo, Marek Bruna

**Affiliations:** 1 Faculty of Mechanical Engineering, Department of Transport and Handling Machines, University of Zilina, Univerzitná 8215/1, 010 26 Žilina, Slovakia; miroslav.blatnicky@fstroj.uniza.sk (M.B.); jan.dizo@fstroj.uniza.sk (J.D.); 2 Faculty of Mechanical Engineering, Department of Applied Mechanics, University of Zilina, Univerzitná 8215/1, 010 26 Žilina, Slovakia; milan.saga@fstroj.uniza.sk; 3 Faculty of Mechanical Engineering, Department of Technological Engineering, University of Zilina, Univerzitná 8215/1, 010 26 Žilina, Slovakia

**Keywords:** aluminium alloy, steering mechanism, vehicle frame, welding, mechanical properties, SysWeld

## Abstract

Nowadays the automotive industry is mainly focused on competition, and this fact forces vehicle producers to constantly look for improvements in the areas of quality and reliability. Life-span, flawless operation, and safety are directly interconnected. Therefore, much attention and resources are spent on research factors that affect the stated properties. Significant capital is invested in the optimization of the constructional solutions and innovative material applications related to the safety and durability of the constructions. This paper presents the results obtained while developing a new ecological three-wheeled vehicle. The main research areas were focused on replacing the original material with a light aluminum alloy, while achieving a substantial improvement in drivability for the three-wheeled vehicle by implementing a modified front wheel steering system. The submitted research achieved a weight reduction of the frame by 40 kg by applying light material substitution (EN AW 6063.T66), which will naturally have a positive impact on the range of the designed electric vehicle; furthermore, we implemented an innovative steering mechanism optimized during the experimental operations.

## 1. Introduction

The frame has been an essential part since the invention of the first automobile. The frame is the basic supporting structure for every transportation means, and its role is to provide a precise and accurate position for the particular construction assemblies to prevent their damage. The frame of each vehicle transfers almost all types of load. As a result, there are significant requirements related to vehicle frames. The frame and its individual parts have an important impact on handling the vehicle, stability, comfort for passengers, and total drivability. It is safety and appropriateness of the construction that play key roles in construction design. In addition to these conditions, the shape, dimensions and material must be considered for the final design to meet all requirements on the strength when designing the vehicle frame. The frame must provide flawless operation of the vehicle with respect to a high degree of security, while also accounting for aesthetics and economic factors. In addition to the above-stated properties, the frame design mostly affects the active and passive safety.

The life-span of the vehicle frame design is directly connected with the fatigue life of the applied material [1]. During vehicle operation, static loads of frames occur occasionally, or not at all. In most cases, frames are exposed to periodical cyclic loads that are the results of driving on an uneven roadway, which causes stochastic excitation [2,3]. The result of the cyclic loading is the existence of the periodically generated loads acting against the frame. After a certain period, these loads may cause microscopic damage to the material; firstly, a gradually cumulative damage and cracking develop, which may result (together with other loads) in the breaking of a component [4,5].

Light materials, such as aluminium and its alloys, are more and more frequently implemented in the vehicle production nowadays. This enables a reduction in the total weight of the vehicle, which is directly connected with the fuel consumption and production of CO_2_ [6]. The mechanical properties of the aluminium alloys are affected by their chemical composition, technological production methods or methods of processing [7,8]. The vehicle frame is a rather complicated construction that requires implementing several technological operations, such as welding or machining.

The main method for connecting the components produced from aluminium and its alloys into one assembly is welding, due to its high productivity and relatively simple application. On the other hand, with respect to the welding of the material, the welding process of aluminium and its alloys is a relatively complicated procedure that significantly affects the final chemical and mechanical properties [9]. To provide reliable long-term operation of the vehicle with an aluminium frame, we need to investigate the ways in which the various welding conditions affect the static strength and fatigue life [1,7].

As a result of the welding process, deformations and residual stress in the welded construction inevitably occur. It is just a relatively low strength, and a relatively large amount of heat that is brought into the material which represent the main reasons for deformations of the welded structures. These negative impacts are strikingly significant in the case of welding aluminium compared with welding of a standard material applied in the frame designs of the vehicles, i.e., steel [10,11]. The shape of the welded surface specifies the state of the welded construction. According to some researchers, this factor may affect residual stress, static strength and welding deformations [12,13].

The laser is more and more frequently utilised when welding aluminium and its alloys. In comparison with conventional methods of welding, a laser beam can generate a large amount and intensity of the heat only in the close area around the weld. This technology, however, is not available, and its application also requires specific conditions [9,12,14,15].

The selected material for the frame design of the three-wheeled vehicle, the aluminium alloy EN AW 6063.T66, was the subject of laboratory tests to find the tensile strength by means of a static tensile test. The test represents a standard procedure by evaluating and verifying the strength characteristics of the materials [12,16,17]. However, in this case, a sample with the weld was tested to measure its impact on the selected material.

Moreover, the presented results of the numerical calculations in the welding process were obtained via the simulation programme SysWeld. This software, applying the finite element method for calculation, is used to obtain knowledge on heat conductivity in the welded material, its deformations during and after the welding process, the origin and extent of the residual stresses [12,13,14]. The programme contains models for a wide range of materials, namely steels, aluminium alloys, and other high-strength materials for complex applications [15,16,18,19].

There exist large numbers of the calculation models and criteria assigned for a prediction of the life-span of elements that are loaded by the uniaxial and multiaxial loads. The present-day state in the sphere of the criteria development for predicting the fatigue life was described by Stefanov [20], the translation cited by Papuga [21]: It is common that each author develops their own criterion for fatigue life prediction and verifies it through the acquisition of experimental data. Then, some other author’s data is not satisfied by that criterion, and a new one is suggested. Thus, too many proposed criteria have been accumulated, and this testifies, in fact, against the approach of reduction. Many criteria have remained isolated from each other, without comparison or competition.

This results from the above-stated ideas that the results of the development of the criteria for the prediction of fatigue life of materials obtained so far cannot be generally accepted. At the same time, we may consider them to be an incentive for the further development.

Many road transport vehicles with fossil fuel engines have a negative impact on the environment, increasing fuel prices, and other largely environmental aspects [6,22,23,24], and thus it is necessary to create solutions that are capable of eliminating such deficiencies to the greatest extent possible. Four-wheeled vehicles tend to be designed for four or five passengers and their luggage. However, this solution greatly limits the possibilities for reducing the fuel consumption. On the other hand, means of transportation such as bicycles, mopeds or motorcycles can be more economical. Their disadvantage is their limited transport capacity, and they do not protect passengers against severe weather conditions. The three-wheeled vehicle represents an acceptable compromise [25,26].

Thus, the authors decided to design an efficient and ecological vehicle, a three-wheeled electric vehicle with one wheel in the front and two wheels in the back. This wheel arrangement has one disadvantage, namely its low stability during cornering [27,28,29]. The designers of three-wheeled vehicles implement various technical solutions to reduce this negative phenomenon [30,31], and to be more specific they use various equipment and devices that enable a change of the vehicle’s centre of gravity, chassis inclination, geometry modification, etc. [32,33]. Such equipment is relatively demanding to control, as it requires the application of actuators [34,35,36], which causes an increase in the complexity of the whole system, energy consumption for its drive, and decreases the reliability. Electric drive-trains use an electromotor with permanent magnets [37], because this type of the drive-train is widespread in electric vehicles.

The presented technical solution enables an increase in stability of the three-wheeled vehicle during cornering, while operating on a purely mechanical principle and, as a result, not employing any additional electrical drives [38].

**Figure 1:**
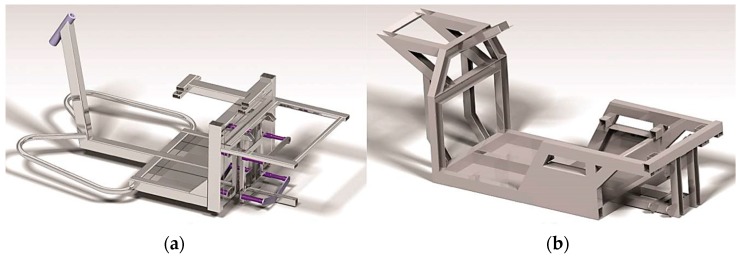
Frame comparison: (**a**) a steel frame of the commercial electrical three-wheeled vehicle; (**b**) a design solution of the frame from the alloy EN AW 6063.T66 for the designed three-wheeled vehicle.

This unique system of steering of the front wheel is mounted in the front part of the frame of the vehicles produced from the investigated aluminium alloy EN AW 6063.T66 (Figure 1b). The drive-train mechanism and power units of the vehicle are mounted in the rear part. In comparison with the conventional steel frame (Figure 1a), the new frame (Figure 1b) is lighter and has a lower centre of gravity. The operation of such a vehicle will be more ecological and safer.

## 2. Materials and Methods

**Figure 2:**
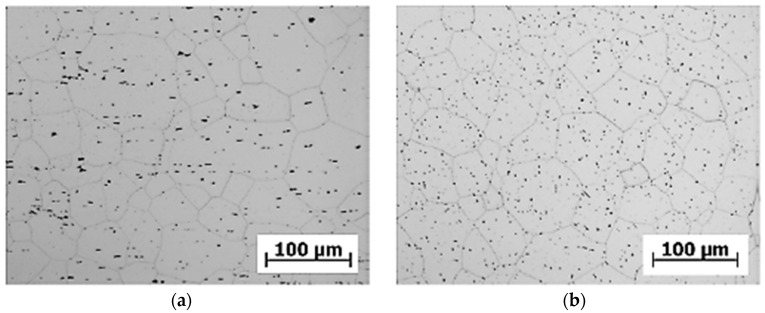
The microstructure of the selected alloy EN AW 6063.T66 in (**a**) the longitudinal direction, and (**b**) the transversal direction, etched by 0.5% HF.

**Figure 3:**
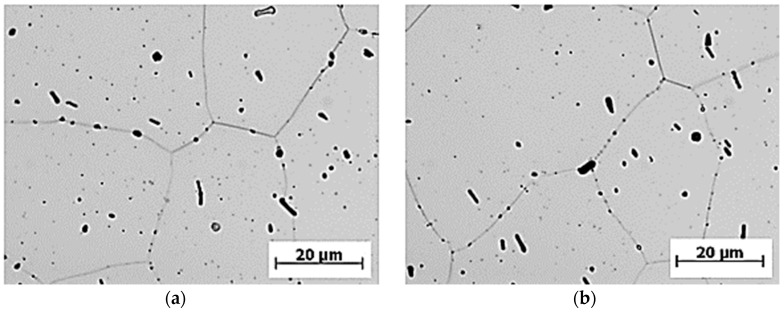
The microstructure of the selected alloy EN AW 6063.T66 in (**a**) the longitudinal direction, and (**b**) the transversal direction, etched by 0.5% HF.

The commercial aluminium alloy EN AW 6063.T66 (Figure 2 and Figure 3) was selected to be the construction material to form the frame of the designed three-wheeled vehicle with innovative front-wheel steering due to its availability and price.

To obtain the required properties of the designed frame, the selected material underwent tests in the laboratory. The tested material was supplied in the shape of a rod with the circular cross-section of 10 mm. The construction material EN AW 6063.T66 was supplied in the compressed state, and therefore there was an assumption of the affected structure caused by combining the mechanical and thermal processing. This assumption was consequently tested by the metallographic analysis, measurement of hardness by the Brinell tests and the static experiment in the tensile tests. The samples were embedded in a mixture of bakelite and dentacryl. The grinding was performed with wet grinding discs in the Struers LaboPol 5 (Struers A/S, Ballerup, Denmark) system, with a gradual application of grinding discs with grains of 160, 300 and 600 with a subsequent cleaning with alcohol and water. Another applied operation was polishing with diamond paste with a granularity of 0.5 and 0.2 μm. The structure of the material EN AW 6063.T66 was revealed by etching with 0.5% HF for 60 s.

As shown in Figure 2 and Figure 3, it can be assumed that the structure of the tested material in the longitudinal and transversal directions is formed by the polyhedral grain of approximately the same dimensions. Uniformly distributed intermetallic phases are observed in the grain of the tested material, shown in detail in Figure 3. The grain boundaries show the presence of segregates. With respect to the distribution of the intermetallic phases and polyhedric grain sizes, we can assume that the microstructure of the material EN AW 6063.T66 does not show any attributes of the state after forming. With respect to the way of the heat treatment, it is evident that the grain recrystallization took place in the process of annealing the solution. We also assume that this was one of the reasons for using the heat treatment.

As it is necessary to connect the parts into more complex systems in the frame structure of the designed three-wheeled vehicle, and also due to the nature of most applied type of joint for vehicle frames, i.e., the welded joint, the complex evaluation of the impact of welding on the fatigue life of the applied material is crucial.

The authors selected a method of welding using non-consumable tungsten electrodes in an inert gas atmosphere, i.e., alternating current TIG.

After specifying the welded material and welding technology, we can propose a suitable filler material and define the optimum welding process. As filler, three materials can be used, namely SG-AlMg_5_Cr(A), AlMg_5_ and AlSi_5_. Due to its availability and usability, the filler material AlSi_5_ was chosen for making welded joints. The parameters (welding conditions) of the welding equipment were as follows:

- welding voltage U_z_ = 18.8 V,
- welding current I_z_ = 79 A,
- filler material AlSi_5_,
- diameter of the filler material Ø = 2 mm,
- diameter of the tungsten electrode Ø_d_ = 2.4 mm,
- shielding gas Ar 99,996% was used with the flow of Q = 15 L∙min^−1^

**Figure 4:**
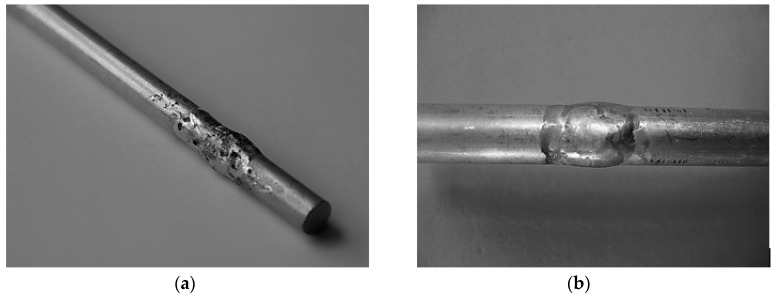
Aesthetics of the welding joint obtained (**a**) without preheating of the basic material, and (**b**) with preheating.

The investigation of the quantification of the welding parameters was necessary due to the stable ignition and burning of the welding arc in the welding samples. Any higher setting of values of the welding current was not possible, as the dimensions of the weld did not permit increasing the high local temperatures. This phenomenon, in combination with the excellent thermal conductivity of the tested material, caused melting of the whole volume of the metal of the sample tested. On the contrary, the lower values of the welding current caused the occurrence of failures of the welded joint, i.e., lack of fusion and incomplete weld penetration. They affected the ability of the material to transfer the force effects or aesthetic failures occurred (Figure 4b).

The methodology used for the test sample formation was designed from the point of view of the impact of preheating of the basic material on the final quality of the welded joint [39]. Based on the generally valid practical recommendations about preheating of the material to be welded, the authors decided to preheat the whole specimen to a value of 150 °C. This temperature was reached by indirect heating of the material, i.e., by the oxyacetylene flame. After preheating, the temperature of the location at which the welded joint was to be made was measured using the pyrometer. This experiment consisted of welding with preheating and without preheating, slow air cooling, machining of the samples according to the required sample geometries for the static test in tension and the comparison of results. The first difference in the quality of the welded joint with the applied preheating could even be recognized visually (Figure 4).

**Figure 5:**
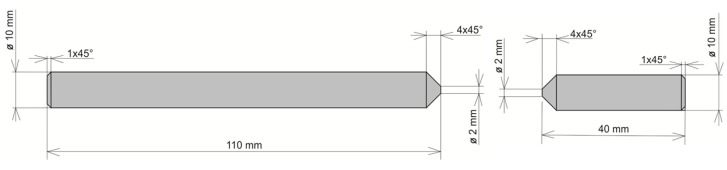
Alpha-geometry of the weld surface for the evaluation of the fatigue characteristics.

The selected indicators of the welded joints measure the weldability of the aluminium construction materials, to be more specific, these were indicators of integrity and tensile strength. With the application of preheating, more stable ignition and burning of the welding arc occurred, resulting in the aesthetics of the welded joint. The aesthetics of the joint is essential, especially in cases where further machining of the joint is not deemed to increase the product price. This is crucial for manufacturing the vehicles. A comparison of the impact of preheating took place in the so-called α-shape weld surface (Figure 5).

**Figure 6:**
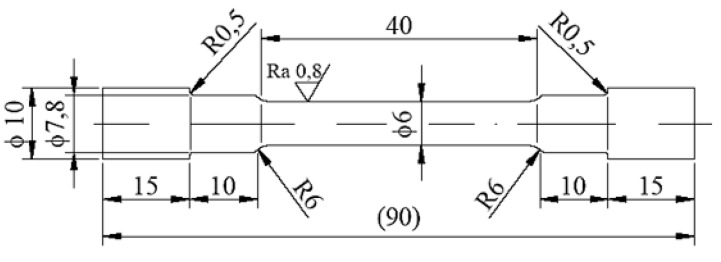
Geometry of the sample for static tension test.

The air-cooled samples were machined following the required geometry (Figure 6) to perform a static tension test. The static tension test was significant in determining the calibration curves for the designed testing conditions.

**Figure 7:**
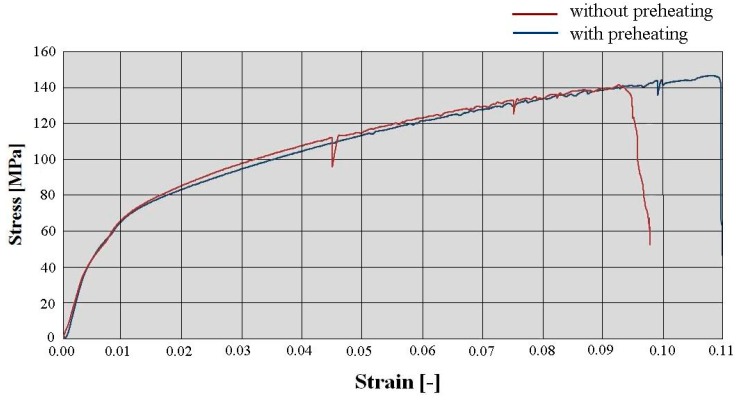
Diagram of the static tension test performed on the welded material EN AW 6063.T66 without preheating (red curve) and with preheating (blue curve) in the alpha-geometry of the weld surface.

Based on the tension diagrams (Figure 7), we can assume that a slight increase of the sample strength (from 142.8 to 146.7 MPa) took place as a result of the applied preheating of 150 °C on the tested samples. The number of the tension tests was 10 for both methods (with and without preheating), and the depicted diagrams are similar to the average values based on their shapes. The result is that the stated graphs serve as documentary material. In most cases, the fracture of the specimens occurred in the welded joints. Fracture initiation was only observed in the melting-down location in occasional cases, but the fracture propagated toward the welded joint.

Before fatigue testing, the value of the current material strength after welding was equal to the static strength of the tested sample. In the process of the fatigue test, degradation of the residual value takes place. The quantity in a specific moment reaches the critical value of the maximum possible load, and this results in the origin of failure. Therefore, it is recommended that all possible precautions leading to an increase in the strength of the welded joint be taken. As a result, the research dealing with the impact of the welding area shape on the welded joint strength was included in the methodology of forming the samples.

**Figure 8:**
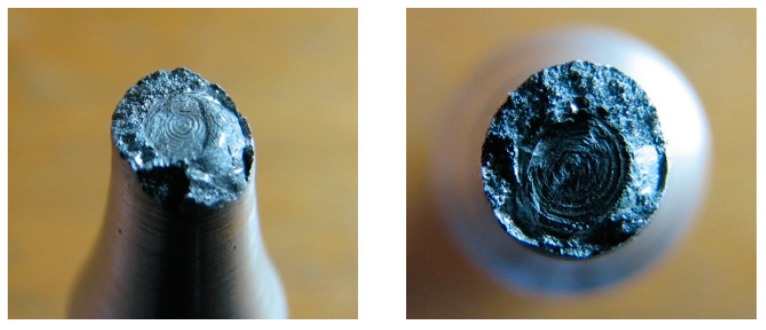
Macrostructure of the fracture areas with the alpha-geometry of the weld surface.

The deficiency of the alpha-geometry (Figure 5) of the weld surface resulted in widespread failures in the welded joint. More specifically, improper welds were created, as shown in Figure 8, and the melting of the weld surface did not take place either. The proof of this statement is the macrostructure of the fracture area on the tested sample, in which we can observe grooving that was caused by the machining tool during the preparation of the welding area. Only the filler material was molten, and its thickness was insufficient after machining in accordance with the geometry of the samples for the fatigue evaluation (or the tension tests). It was formed by the thick ring of the filler material, which was softened by welding, so we cannot speak of sufficient joint strength.

**Figure 9:**
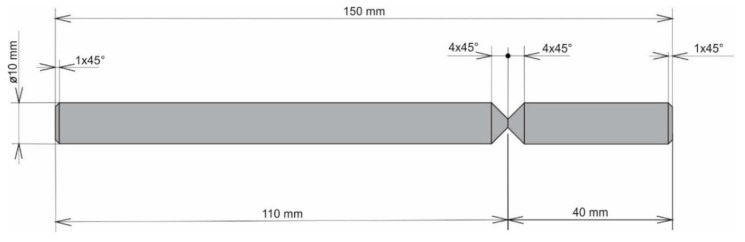
Beta-geometry of the weld surface for evaluation of fatigue characteristics.

Removal of the failures of this origin would be relatively easy with more extensive proportions of the welded material. Increased values of the welding current would become a solid part of the technological production method. However, as stated above, this method is not suitable, as an increase in the thermal areas emerges, causing melting of the whole volume of the samples. Therefore, we modified the geometry of the weld surface. It is evident that for the fatigue tests, only the shape of the weld surface that showed the highest strength was applied. As a result, the original shape of the weld surface was modified into a new one, the so-called beta-geometry (Figure 9).

**Figure 10:**
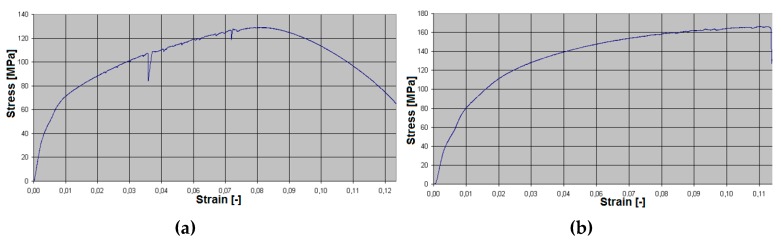
The diagram of the static tension test performed in the welded material EN AW 6063.T66 with preheating (**a**) in the case of the beta-geometry of the weld surface, and (**b**) in the case of gamma-geometry.

**Figure 11:**
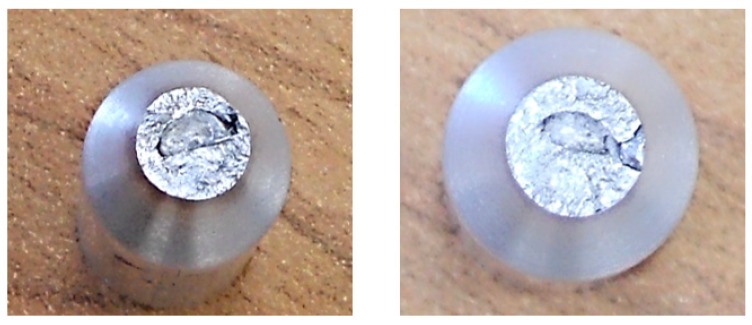
Macrostructure of the frature areas with beta-geometry of the weld surface.

A formation of a weldment from one piece (this would be a problem in designing the vehicle frame, but a possible increase of welding current could be the solution) led the authors to consider the beta-geometry of the weld surface. The formation of the weldment, the modified weld surface was subjected to cooling in the same conditions as applied in the previous case. After welding and cooling, machining of the sample followed, and the final shape underwent static tension testing (Figure 6). The obtained graph serves as proof (Figure 10a). The tensile strength after the beta-geometry of the weld surface decreased on average. The value of the strength of ten samples was 129 MPa, which means a strength reduction by 12% on average against the alpha-geometry of the weld surface. By observing the macrostructure of the fracture (Figure 11) of the modified weld surface after welding and in comparison to the previous macrostructure, we found out that a certain change had taken place. The change did not result in a sufficient welding connection. In the welded joint, inclusions frequently emerged—these were the result of mixing the welding metal with the oxide layers Al_2_O_3_ while melting the metal connecting both parts of the weldment. Therefore, it was also necessary to request a modification of the weld surface geometry of the tested sample.

**Figure 12:**
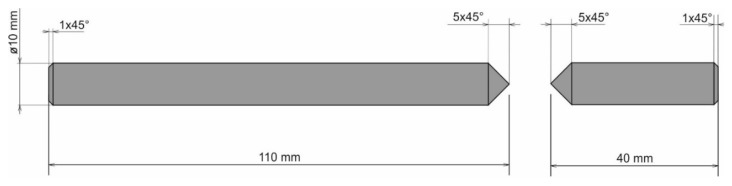
Gamma-geometry of the weld surface for the evaluating the fatigue characteristics.

Another design of the weld surface geometry of the sample (Figure 12) follows the assumption that removing the connection of the base material located at the weldment could eliminate the formation of oxide-based inclusions. At the same time, the elimination of the blunting level between welding parts ensures complete penetration across the entire thickness of the weld.

The welding of the samples was performed by implementing the same welding parameters as in the previous two cases. After cooling and machining into the final shape, the static tension test followed, and its diagram is presented in Figure 10b. The tensile strength arithmetically reached a value of approximately 165 MPa, which is an increase of 30% on average compared with the value of the strength after welding a given type of the alloy, as stated in [39], which is 100–120 MPa with the usage of the same filler material.

**Figure 13:**
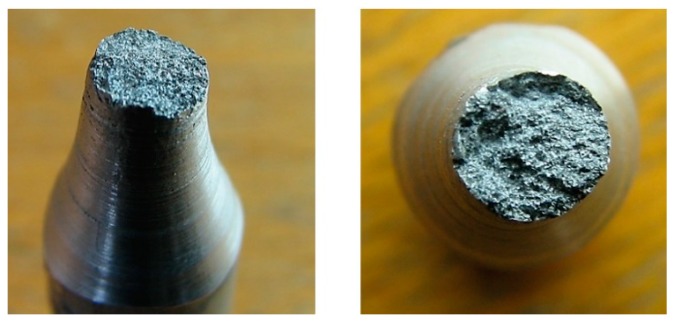
Macrostructure of the fracture area with gamma-geometry of the weld surface.

The stated value of tensile strength set by the static tension test for the basic material EN AW 6063.T66 is 247 MPa. That means that, due to the welding process, the strength decreased to 70% of the original value during welding with the gamma-geometry of the weld surface. This geometry; however, showed the highest strength after welding from all geometries applied, and this was the reason for its selection for testing of the welds in the multiaxial fatigue tests. The same shapes of the weld surfaces will be applied in the frame development of the designed three-wheeled vehicle. The macrostructure of the fracture area of the gamma-geometry is shown in Figure 13. The macrostructure does not manifest the presence of defects caused by welding. The achieved quality and strength of the weld are sufficient for the implementation in practice, and the measurement of the fatigue characteristics of the material are conducive to the possible implementation of the material in the frame structure of the designed three-wheeled electric vehicle.

## 3. Application of Simulation Software

The reason for implementing numerical simulations of welding in practice is the definition of individual components’ deformations and the investigation of the possibility of failure formation. All of this can be foreseen on the basis of certain parameters, such as material structure, hardness, residual stress, and total plastic deformation [10,12,13,40].

**Figure 14:**
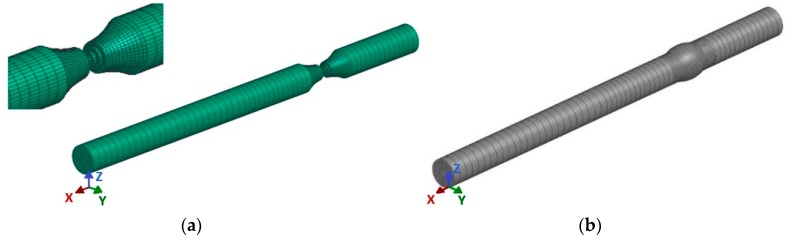
The formation of (**a**) weld surface gamma-geometry and (**b**) the 3D model of the weldment sample, designed in the software SysWeld.

The first step in solving the issue was the formation of the finite element model (FE model) of the sample’s weld surface (Figure 14a). The software SysWeld 2017 (ESI Group, Paris, France) was applied to solve the task. It is among the world-class programs for complex solutions of welded joints.

**Figure 15:**
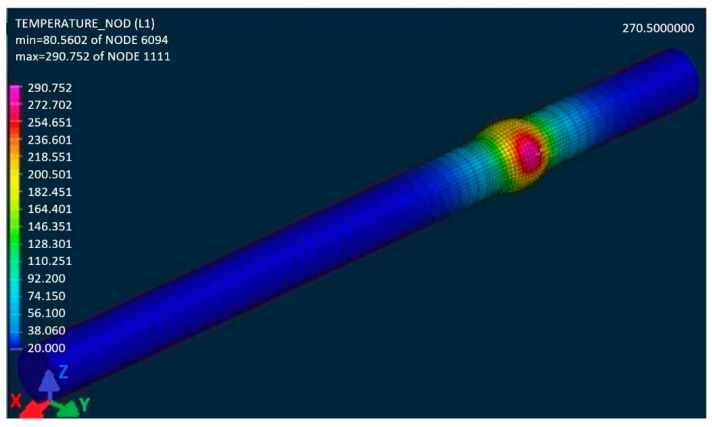
The simulation of the heat gradient of the material EN AW 6063.T66 welded by the TIG technology when implementing the predefined welding parameters for the period t = 0.5 s of the welding time.

**Figure 16:**
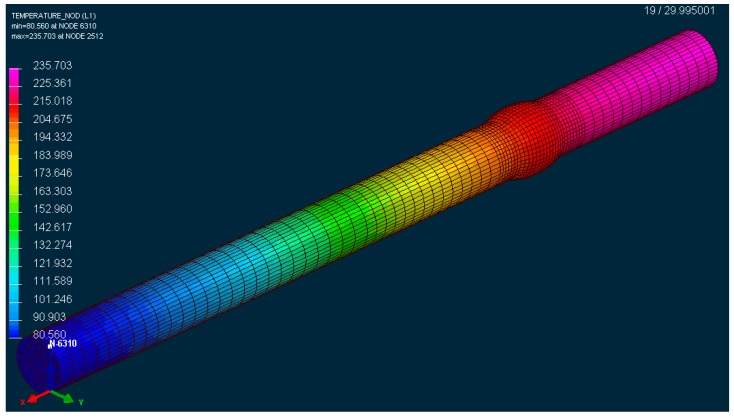
The simulation of the thermal gradient of the material EN AW 6063.T66 welded by the TIG technology with implementing the predefined welding parameters in the period t = 20 s since the end of the welding procedure.

A problem would probably emerge in generating the FEM network if we intended to maintain the identical shape of the weld surface as it was applied in practical welding (gamma-geometry) in the course of the numerical calculations. Non-desirable acute angles would definitely appear in individual elements. Therefore, a virtual modification of the shape of the weld surface was designed (detail, Figure 14a) that does not have any impact from a technological standpoint on the formation of the welded joint compared with the real shape of the gamma-geometry. After designing such a model, we performed the simulation of welding the sample (Figure 15 and Figure 16) with simulated boundary conditions corresponding to real welding (material, sample clamping, welding parameters, etc., without preheating, i.e., the temperature of the welded material was set at 20 °C). The material temperature in the contact of the electrode with the welded material in the period t = 0.5 s reached its maximum value of 288 °C (Figure 15). In the course of the next 2.5 s, the maximum temperature at the point of welding reached a value of 670 °C, i.e., the melting temperature of the tested material. The temperature at the shorter end of the sample was still relatively low, approximately 150 °C. The heating process at the ends is indirect, i.e., it arises due to the heat conductivity in the material. Conduction depends on the coefficient of the heat conductivity λ. There is no direct dependence between conduction and the amount of the alloying additives in an alloy. The higher the amount of the alloying additives in the alloy is, the more the heat conductivity of the material drops. The aluminium itself is a good heat conductor that is non-desirable in this case, as it overheats, affecting the structure of the whole sample volume during welding. The period of welding, as well as the simulation of sample welding, in both cases was approximately 5 s.

The gradient of the obtained temperatures is significant for evaluating the scale of the affected area by heat. This plays an essential role in the results of measuring hardness of individual welded joint components (Basic Material, Weld Metal, Heat Affected Zone).

The result of the welding simulation is the FEM model of the welded sample (Figure 14b), where the analysis of the individual phases’ modifications (material) by heat is carried out, i.e., during the welding process the so called phases (material) were changed as a result of the conducted heat and the consequent difference in the chemical composition of the basic and filler materials. The material can have a different distribution during welding or cooling. Therefore, it was necessary to investigate this state.

**Figure 17:**
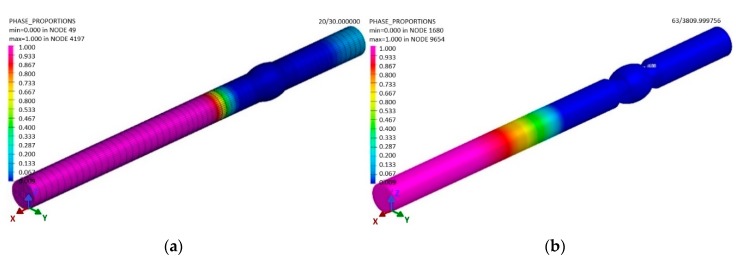
The distribution of the basic material (Phase 1) in the sample (**a**) in the period t = 25 s after welding and (**b**) after cooling in the period t = 3,600 s, according to the simulation designed in the software SysWeld.

**Figure 18:**
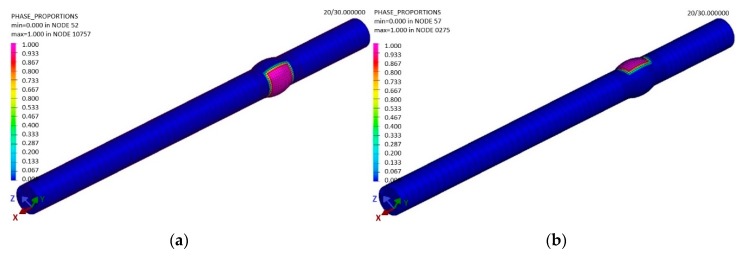
The distribution of (**a**) the welding metal of the first weld bead (Phase 2) at the end of the sample welding in the period t = 30 s and (**b**) the welding metal of the second bead (Phase 3), according to the simulation designed in the software SysWeld.

The applied term Phase 1 represents the percentage of the abundance of the basic material (pink 100%, indigo 0%) that was not affected during welding by heat (Figure 17). After cooling the welded material to a temperature of 20 °C, the software showed a modification in Phase 1. This was caused by the fact that the weld bead still had a high temperature after welding (Figure 16), which was then distributed within the material due to conduction. This resulted in the impact on the material (Figure 17b). Practically, the welded joint was formed by two weld beads in the shape of a semicircle. Phase 2 represents the abundance of the welding metal (Figure 17a) and its impact by heat in weld bead No 1. A similar simulation was performed for Phase 3 (Figure 18), as the weld bead is formed by the same technology as the first one.

**Figure 19:**
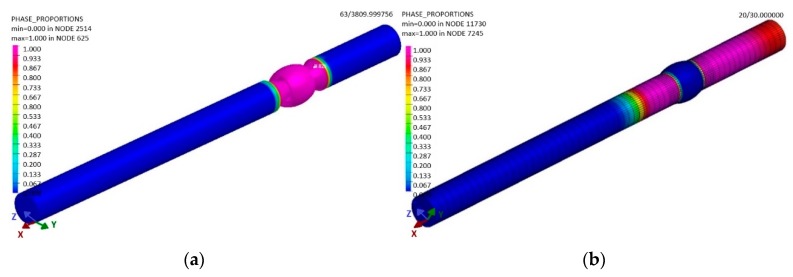
The distribution of the filler material in the sample (**a**) after cooling and (**b**) the heat distribution in the affected area in the period t = 30 s, according to the simulation designed in the software SysWeld.

After cooling, the abundance of Phases 2 and 3 (the welding metal) is as shown in the sample—see Figure 19a.

The final phase in the sample was that formed by the impact of welding is the heat affected area, Phase 4 (Figure 19b). Phase 4 is the area that was not molten as a result of the conducted heat from welding, but, at the same time, where the heat area was so large that a structure modification took place.

**Figure 20:**
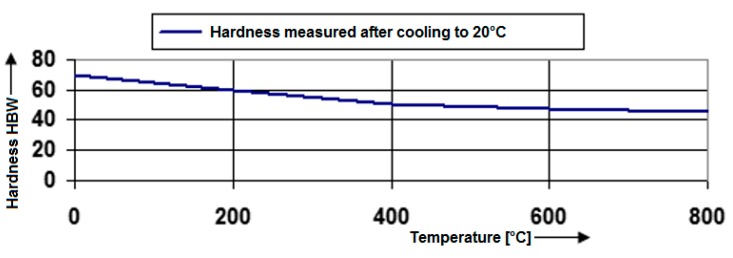
Hardness dependence of the material EN AW 6063.T66 based on the heating temperature during welding.

The impact of the individual phases on the material hardness was investigated by hardness tests. The hardness value of the tested material EN AW 6063.T66 was defined by Brinell when the pressed ball from the hardened alloy with the diameter of 5 mm was pressed into the material during the time of 10.5 s and by the force of 225 N. This hardness test is marked as HBS. In addition to the hardness measurement of the basic material, the hardness measurement of the affected area and the welding metal also took place. Their distribution was monitored by means of numerical simulations. The hardness tests of the basic material, the heat affected area and the welding material showed softening of the material (Table 1, followed by Figure 20) as a result of the introduced heat against the basic material.

The comparison of the hardness of the basic material with the weld is more complicated, as a modification of the chemical composition takes place due to the filler material (the alloy AlSi_5_), and this will result in different hardness values. As the welded joint is not the final shape of the sample, it must be mechanically machined to the required geometry, since the initiation and spreading of the fatigue breakage takes place at the selected point. In this case, it is at the point of the welded joint. Therefore, the machining of the sample was carried out, as this could affect its hardness. The impact of machining can be partially recorded in such a way that surface hardening appears to be a result of machining, which could increase the fatigue life. As a result, it is necessary to measure the micro-hardness in the surface area and inside the welded joint (Table 2).

**Figure 21:**
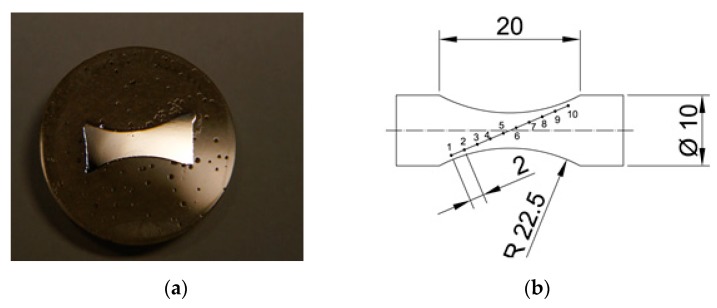
(**a**) The sample for monitoring the microstructure of the welded joint; and (**b**) the schematically described position of the measurement of micro-hardness HVM corresponding with Table 2.

The measured values show a slight increase in micro-hardness immediately below the surface area in comparison with the centre of the sample (Figure 21). The entry can have an impact on the increase of fatigue durability of the samples, but not compared to a non-welded sample, as this is also mechanically hardened by the machining processes.

**Figure 22:**
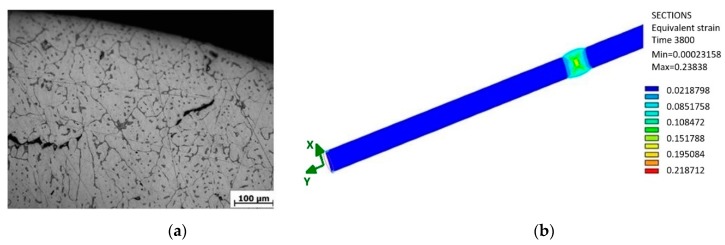
(**a**) The microstructure of the welded joint of the sample in the cross-section with the application of the filler material AlSi_5_, etched by HF 0.5% and (**b**) the strain state in the specimen during twisting stress.

The microstructure of the obtained welded joint in the cross-section is shown in Figure 22a. In the picture, one can see the presence of failures caused by welding. They are cavities that are the result of activities of the gases resolved in the weld pool at high temperatures. During solidification and cooling, the gas does not attempt to diffuse from the weld metal into the surrounding atmosphere, and its presence is manifested in the weld by the presence of a micro-cavity. If such a micro-cavity has greater dimensions, it may act as a fatigue crack initiator. Thus, a faster breakage of the sample takes place.

The position of the failure in the sample cross-section plays a significant role. Analogically, a failure close to the sample surface induces a faster breakage. In the micro-structure, the lightest part is most abundant. It represents the dendritic structure of aluminium in the molten state (during welding the material melted, thus modifying its structure). However, it is more globular, which is explained by a higher speed of cooling due to the small volume of the molten metal. The result is that the metal is more rigid. This fact was proven by the static tension test of the welded sample when a difference between the experimentally measured and theoretical table values from 40 to 50 MPa emerged. Black bodies are failures of the welded joint. Their presence in the samples is stochastic, and cannot be reliably recorded in the process of the fatigue measurement, as each welded joint is unique with respect to its properties. We may determine only based on a certain degree of probability how many pieces of the samples from a certain amount will be insufficient when the amount, size and position of such failures are inadmissible. As a result, it is necessary to monitor the fracture area of each sample after testing. By monitoring the micro-structure, we can observe failures originating very close to the surface of the tested sample. Cavities will merge and spread with a high probability in further research, and thus a crack of greater dimensions will take place, and this will result in a fast fracture.

**Figure 23:**
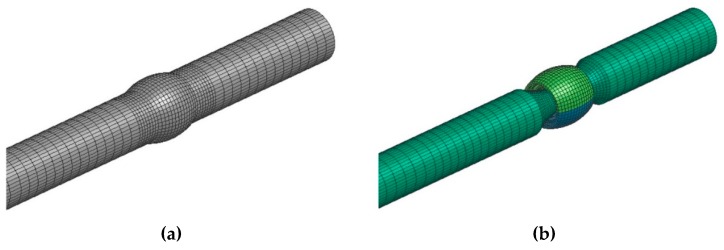
(**a**) The 3D computational mesh model of the formed welded joint; and (**b**) the volume transparent description of the removed material with the aim to produce the sample for evaluating the multiaxial fatigue of the welded joint.

**Figure 24:**
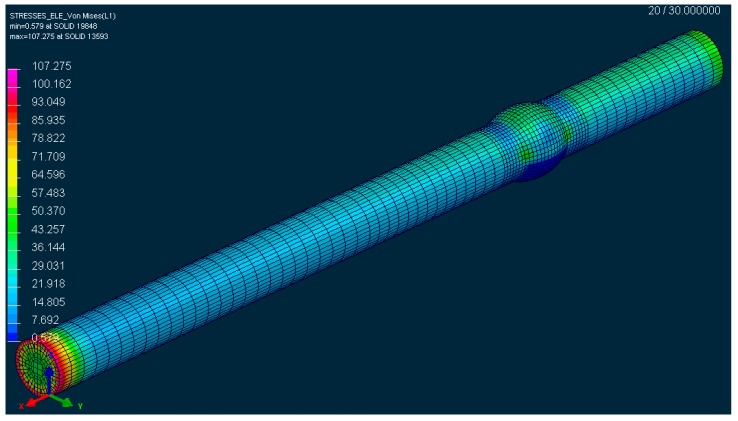
The reduced stress according to the hypothesis HMH in the tested sample after welding in the period t = 30 s, according to the simulation designed in the software SysWeld.

The authors assume that smaller values of amplitudes will not lead to this negative effect and thus, this effect will not appear in such a short period. Therefore, the resistance against fatigue crack will converge gradually, with a decreasing deviation towards the resistance of the unwelded sample, in which, naturally, such failures from welding are not present. Another opportunity of the assumed difference of the fatigue life of both samples is also an impact of residual stresses in the sample that may develop after welding. As the weld was mechanically machined, a material removal (Figure 22b and Figure 23) and a residual stresses removal took place. On the other hand, the chip machining process brings into the material certain the residual stress. Because of combination of these factors, value of the residual stress cannot be precisely quantified. In general, we may assume that the tensile residual stresses degrade the fatigue life, compressive residual stresses, on the contrary, improve fatigue life. The value of the residual stresses from welding was shown by further simulation of sample welding (Figure 24). The performed simulation of welding the sample illustrates a real technological procedure of the sample welding.

The real welding was performed by the position of the sample in the notch. The connected components were attached and locked by collets. By the impact of such sample mounting, the residual stresses are the lowest ones exactly at the point of the stress concentrator. This may be a certain advantage, as in the fatigue process at the point of the sample neck. There, the lowest possible residual stress from the whole sample length with be acting. We measured the value of the residual stress by the simulation following the hypothesis HMH, i.e., approximately 40 MPa. The authors assume that in the low amplitudes of deformation (the complex methodology of evaluating the suitability of the alloy EN AW 6063.T66 also consists of the formation of a real multiaxial testing device with the controlled amplitude of deformation), the sample will resist the fatigue process better due to the residual stress. On the contrary, in the high amplitudes of deformation, it is expected that an impact of residual stresses will quickly reset and thus it will not have any significant impact on the fatigue life of the sample. In practice, it means that in this case the residual stresses caused by welding will affect the fatigue life of the material favourably (by low amplitudes of deformation) or they will have a minimum impact (by high amplitudes of deformation).

## 4. Construction Design of Innovated Mounting of the Front Wheel

**Figure 25:**
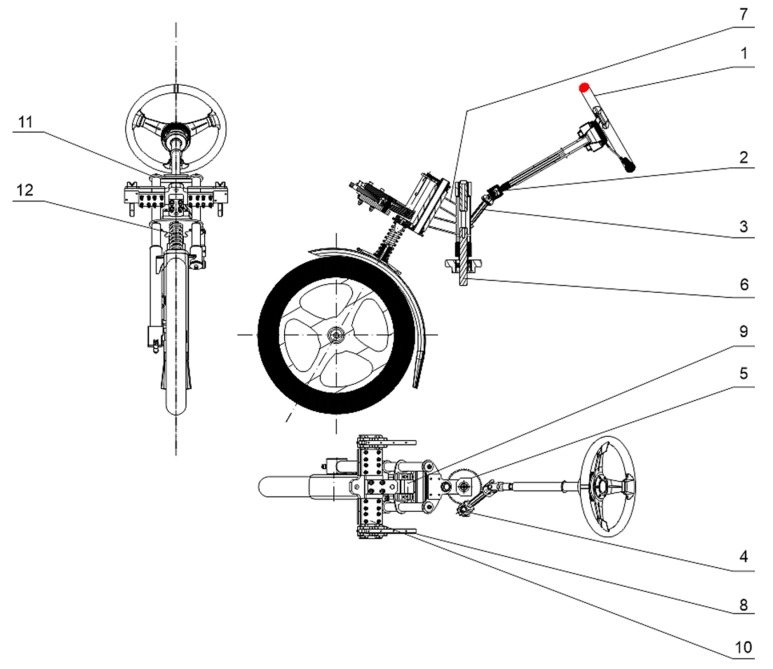
Draft, side view and footprint of mounting the front steered wheel for the three-wheeled vehicle.

The presented technical solution deals with a construction solution of the mounting of the front wheel of the three-wheeled vehicle with the aim to increase the stability of the vehicle. These days, the conventional conceptions of the mounting of the steering wheel pivotally mounted in the vehicle frames are applied to steer bicycles, mopeds and motorcycles. This construction solution, however, also provides a set of disadvantages resulting from the static behaviour of the contact point of the steered wheel with the road in the conception with the steered front wheel. Although the rotation of the wheel takes place to the left and right, the position of the contact point is almost permanent which has a considerable impact on the stability of the three-wheeled vehicle for driving in the curve [41,42,43]. The solution of the stated issue consists of the mounting of the front wheel fork into a rotary of a mounted element and a joint. Based on the knowledge, a requirement for forming such a conceptual solution applied in the three-wheeled vehicle with one steered wheel in the front and two wheels in the back arose, which will provide a stability increase during driving through a curve in connection with the driving safety, especially during deceleration. There is also some space for solving the problem using suitable technical means. The design of a construction solution for mounting the front steered wheel of the three-wheeled vehicle (Figure 25) is the result of this effort.

To steer the wheel, a steering wheel is used instead of the traditional handlebars (1). The steering column contains the output in the form of a cardan shaft (2), which is pivotally mounted in its guidance at the opposite end. Consequently, a transfer of power into the system is guided by a cardan shaft (3) connecting the shaft end with a driving toothed pinion (4). The pinion is engaged with a great driven toothed wheel (5) with a gear ratio of 1:3. This enables the vehicle to be steered comfortably, which is necessary especially in the cases when the wheel returns to a straight direction. There is a console (7) rigidly mounted to the shaft of the great toothed wheel (6). Therefore, during turning the wheel, a rotation of the console takes place, but with a modified sense of rotation. In the front part of the specifically constructed frame, there is a linear guidance (8) mounted on both sides which provides a component of the movement of the fork in the direction of the vehicle length. From the rotation results, the cross-section component of the vehicle movement is provided by the console that has the function of the fork carrier.

The fundamental sign of the mounting of the steered front wheel is that in the longitudinal axis of symmetry of the linear guide system there is a rotating mounted rod (9) performing a direct reciprocating motion in the direction of the linear guide system and the rotation motion that is carried out by the fork and provided by the console. The rod is bolted into the suitable moving plate (10) that connects the wheel fork (11) with the console. With the length of the rod of 75 mm, the angle of rotation is 40°, and that means, in practice, that the rotation of the vehicle in the curve has a radius of 1.5 m. Meanwhile, due to comfort sake, the front fork is equipped with the suspension system consisting of the spring and damper (12). The advantages of mounting the steered front wheel of the three-wheeled vehicle like this are distinct from the direct effects.

**Figure 26:**
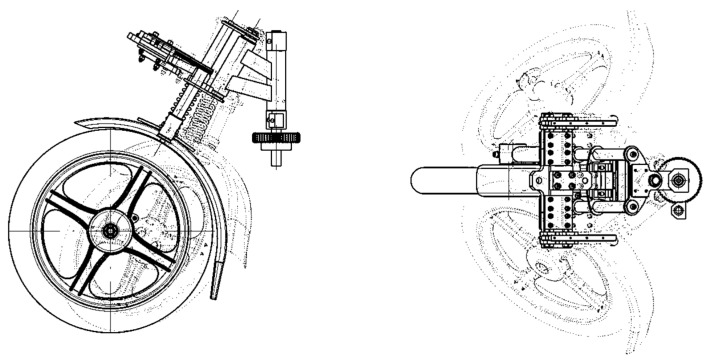
The possibilities of moving the front wheel enabled by a new way of the designed arrangement with the aim of increasing the stability of three-wheeled vehicles during cornering.

**Figure 27:**
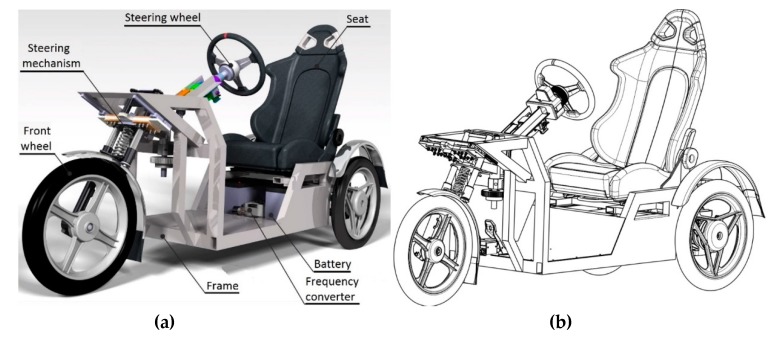
(**a**) The 3D CAD model of the designed vehicle and (**b**) the schematic drawing of the three-wheeled vehicle with the non-conventional mounting of the steered front wheel with the frame from the tested material EN AW 6063.T66.

**Figure 28:**
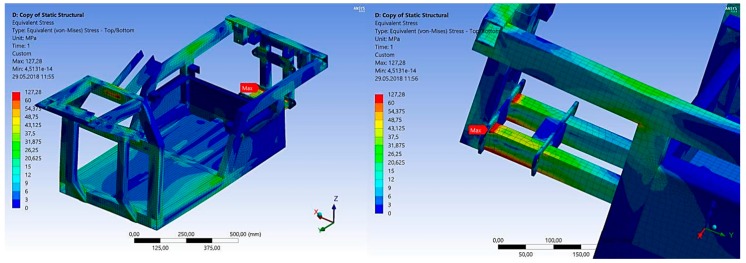
The simulated stresses in the material EN AW 6063.T66 of the frame of the designed vehicle in driving through the curve with the aim of defining the central stress as an independent entry for evaluating the fatigue life, the simulation designed in the finite element software ANSYS 17.2 Workbench.

In general, we may assume that the originality of the submitted device consists in the fact that the designed unique device is able to increase the stability during driving though a curve in connection with keeping the safety standards, especially during deceleration. The stability of the three-wheeled vehicle is increased by a non-conventional mechanism of the wheel steering. This enables a wheel rotation, as well as its twisting and inclination (Figure 26). For a given technical solution, a system of its mounting in the vehicle frame (Figure 27) made from a tested commercial alloy of aluminium EN AW 6063.T66 was designed. When forming the frame of the designed vehicle, its shape, dimensions and materials had to be considered so that the final product would meet all strength conditions. The drive unit of the three-wheeled vehicle is mounted in the rear part of the designed frame. This means that the part is modified for an engine to be positioned as well as a differential gear, suspension, etc. The frame was designed from the aluminium alloy material EN AW 6063.T66 with the tensile strength Rm = 247 MPa and yield strength Re = 140–160 MPa. In the construction of the frame, we paid our attention to the greatest possible application of standardized aluminium profiles. The static tension tests were performed to measure the total rigidity and load of the construction of the designed frame (Figure 28).

**Figure 29:**
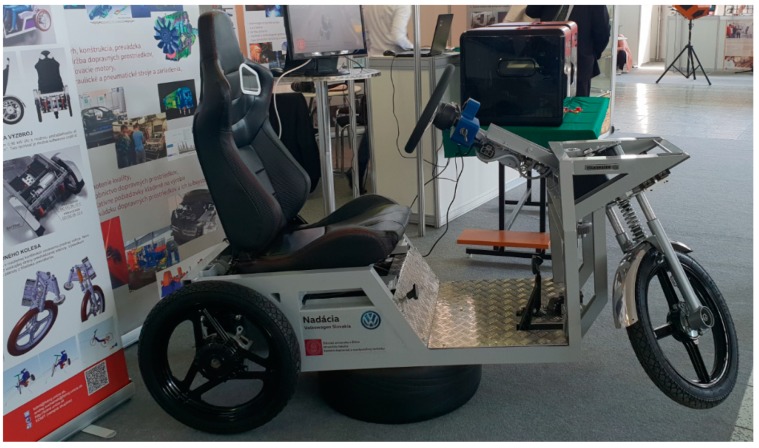
Real model of the designed vehicle.

In the analysis, we investigated the points at which the concentration of stresses, as well as the intensity of the stresses and shifts resulting from loading forces, took place. Two boundary conditions had to be considered in the static analysis. The first condition takes place when the designed vehicle is at rest or in the linear motion, and there are no acting lateral forces developed by the motion in the curve. The second boundary condition takes place in the crossing of the curve with the regarded lateral acceleration of 0.5 multiplication of the standard gravitational acceleration (g = 9.81 m·2s^−2^). The construction design of the frame E3-cycle was created according to the technical standards and rules of designing with the implementation of knowledge obtained during the previous simulations. The numerical strength analysis was used to analyse all possible states of the vehicle in real handling. In the designed vehicle (Figure 29), no devices initiate excited vibrations that would cause system resonance, thus damaging the frame, which secures its strength and structural integrity.

The future research in this field will be focused on analysis of dynamic properties of this vehicle in multibody software, e.g., Simpack. Our effort will include the created FE model of a frame into a multibody model of the vehicle and there to analyse the response of the mechanical system of the vehicle under loads [44,45], which simulate realistic driving conditions.

## 5. Conclusions

The main aim of the authors was the effort to form a complex methodology serving for designing a three-wheeled transportation means with a new type of the front wheel handling. The frame of the original construction of the three-wheeled vehicle was produced from the construction steel. The submitted research suggested a light material substitution (EN AW 6063.T66) ensuring a weight reduction of the frame by 40 kg, which will naturally have a positive impact on the range of the designed electric vehicle.

The design modification, which consists of lowering the seat arrangement for the driver by 150 mm, as provided by the application of the tested aluminium alloy, will have, as the authors assume, a positive effect on the driving characteristics of the vehicle which will be monitored in the further research of this complex topic through implementation of the MBS software.

## Figures and Tables

**Table 1:** The measurement of sample hardness.

Point of Hardness Measurement					
Basic Material		Heat Affected Zone		Weld Metal	
Number of Measurement	Value of HBS	Number of Measurement	Value of HBW	Number of Measurement	Value of HBS
1	61	1	52	1	46
2	69	2	46	2	48
3	65	3	50	3	48
4	66	4	51	4	48
5	66	5	50	5	47

**Table 2:** The measurement of micro-hardness of the welded joint according to Vickers (HVM).

Number of Measurement	1	2	3	4	5	6	7	8	9	10
HVM	69	61	57	56	50	56	54	59	60	63
